# The effect of clinical pharmacists’ intervention in adverse drug reaction reporting: a retrospective analysis with a 9-year interrupted time series

**DOI:** 10.1186/s12913-022-08320-8

**Published:** 2022-07-19

**Authors:** Tianwei Lan, Hua Wang, Xin Li, Hang Yin, Dan Shao, Yueyao Jiang, Qian Yu

**Affiliations:** Department of Pharmacy, China-Japan Union Hospital of Jilin University, Changchun, China

**Keywords:** ADRs reports, Clinical pharmacists, Interrupted time series, Intervention, Unreported ADRs, Standardized reporting rate

## Abstract

**Background:**

In China, 85.4% of adverse drug reactions (ADRs) are spontaneously reported by healthcare facilities. As a result, many ADRs are not reported due to lack of mandatory reporting requirements. As healthcare professionals, clinical pharmacists (CPhs) serve as a bridge between clinical work and medication and ensure rational drug use. In China, A team of CPhs implemented an intervention for ADRs reporting, with the goal of improving the number of ADRs reports, the number of unreported ADRs, and the standardized reporting rate.

**Methods:**

On June 01, 2015, a team of CPhs implemented an intervention for ADRs reporting at a Grade A, Class 3 hospital in China. The drug review catalogue (DRC) was used to screen physician orders for having visible symptoms of ADRs across departments, pooled the ADRs, and submitted them to the Center for Advanced Drug Monitoring (CNCAM). We retrospectively analysed the effect of a CPhs ADRs reporting intervention on the number of clinical ADRs reports, the number of unreported ADRs, and the standardized reporting rate over a 9-year period by interrupted time series (ITS). The method was implemented at the hospital on June 1, 2015, and a segmented regression model was used to analyse the data from January 1, 2010, to December 31, 2019.

**Results:**

After the CPhs ADRs reporting intervention, the number of inpatient ADRs reports submitted to the CNCAM immediately increased by approximately 63 (62.658, *P* < 0.01) and then decreased by approximately 1 (0.701, *P* = 0.000151 < 0.01) per month afterward; the number of unreported ADRs was immediately reduced by approximately 44 (44.091, *P* < 0.01) and remained largely unchanged over time (*P* > 0.05); the standardized ADRs reporting rate per month immediately increased by 63.634% (*P* < 0.01) and remained largely unchanged over time (*P* > 0.05).

**Conclusion:**

The CPhs ADRs reporting intervention had an immediate effect on improving ADRs reporting, which highlights the severity of ADRs underreporting in Chinese hospitals. The method is practical and should be used more widely in clinical practice. For example, the method can adjust and establish a DRC catalog that meets the actual situation of the implementing hospital based on the hospital's drug use habits and has the characteristics of good adaptability. However, it does have some limitations; for example, it may be difficult to detect early ADRs without visible symptoms.

## Background

The concept of drug safety/pharmacovigilance is raising awareness of the various effects of drugs [1]. The establishment of adverse drug reactions (ADRs) databases to collect ADRs reports and evaluate the safety of marketed drugs is important to help healthcare professionals learn more about drugs and ensure their safety. In China, ADRs are primarily reported by healthcare facilities every month via the spontaneous reporting system (SRS) [2]. Although the SRS is an ADRs reporting platform, it cannot spot intentionally or inadvertently missed reports. Moreover, the content and quality of ADRs reports are subject to the reporter's knowledge and background, clinical experience, and patience. In fact, studies show that approximately 10% of inpatients have treatment-related ADRs each year, but less than 5% of these are reported. The actual number of ADRs is much greater than the number of reported ADRs [3]. The China National Centre for Adverse Drug Reaction Monitoring (CNCAM) has three main sources of ADRs reporting, which are medical institutions, drug distributors, and drug manufacturers [4]. In 2020, the CNCAM [2] received a total of 1.676 million ADRs reports, of which 85.4% were spontaneously reported by healthcare facilities [5]. ADRs reporting relies on individual reporters, and no mandatory reporting laws or regulations have been established [6]. In the current healthcare system in China, tens of thousands of ADRs could go unreported each year unless hospitals actively report them [7].

Clinical pharmacists (CPhs) review unusual medication records during treatment and promptly communicate with clinicians to verify ADRs under-reporting, thereby improving the rate, timeliness, and accuracy of ADRs reporting [8]. On June 1, 2015, we implemented an ADR reporting method with CPhs intervention at our hospital, in which a team of CPhs detected ADRs among hospital patients and promptly reminded clinicians to report them. In cases of potential ADRs, CPhs assessed causality, verified the event with clinicians, and reminded the clinicians to report any missed ADRs. In addition, CPhs reviewed ADRs reports for missing items (required items include a personal history of the ADRs, family history of the ADRs, lifestyle factors, medical history, concomitant medications, description of the ADRs [including symptoms, signs, clinical tests], measures taken to treat the ADRs, improvement or resolution of the ADRs after drug discontinuation or dose reduction, ADRs recurrence after the resumption of the suspected drug, and causality) to reduce potential under-reporting and omission and promote the standardization of ADRs reporting. In this paper, we introduce an ADRs reporting method with CPhs intervention that was implemented on June 1, 2015, in a Grade A, Class 3 college teaching hospital in China. Using an interrupted time series (ITS), we retrospectively analysed the number of inpatient ADRs submitted to the CNCAM per month, the number of unreported ADRs per month, and the standardized ADRs reporting rate per month in 2010–2019 to investigate the immediate and long-term effects of CPhs intervention on ADRs reporting.

## Methods

### Study design

A retrospective observational study was performed, and a drug review catalogue (DRC) was established as follows: The CPhs (1) developed a catalogue of rescue drugs for ADRs according to the World Allergy Organization Guidelines for the Assessment and Management of Anaphylaxis [9], Anaphylaxis: Guidelines from the European Academy of Allergy and Clinical Immunology [10], and anti-allergic drugs current used at our hospital; (2) analysed the pattern of medications concomitantly used for ADR treatment and developed a catalogue of adjuvant rescue drugs for ADR treatment (excluding anti-allergic drugs); and (3) combined the two catalogues to calculate the rate of concomitant medication use with anti-allergic drugs and adjuvant drugs, select the drugs used in various combinations, and finalize the DRC based on eligible ADR reports and medication records in the CNCAM.

### Setting

The study was conducted at a Grade A, Level 3 general hospital (the highest level in the Chinese hospital system) under the National Health Commission in Northeast China. The classification of hospital grades in China is based on China's *current Hospital Classification Management Measures,* which classify hospitals into levels 1, 2, and 3 by assessing their functions, missions, facility conditions, medical technologies, and scientific capabilities. Level 1 hospitals are primary health care institutions that provide preventive, medical, health care, and rehabilitation services directly to the community; Level 2 hospitals are regional hospitals that provide health services to multiple communities; and Level 3 hospitals are large general hospitals that provide medical and health services across regions, provinces and cities, as well as nationwide, and are responsible for providing business and technical guidance and personnel training to the above two levels of hospitals. As one of the highest-ranking medical institutions in the "three-tier and six-level" Chinese hospitals, our hospital also undertakes the task of university teaching. The hospital has 2950 beds and discharges 132,200 patients per year.

### Implementation of the intervention

To implement the method, the CPhs used the DRC to screen physician orders across departments, pooled the records of the use of drugs in the DRC, and assessed causality (excluding DRC drugs used to treat nonallergic conditions). Consecutive medication records for the same anonymous patient (case number taken here) are also consolidated into one report to avoid duplicate reporting. In cases of potential ADRs, CPhs reminded the clinicians to report any missed ADRs.CPhs also reviewed ADRs reports for missing items [including symptoms, signs, and clinical tests], measures taken to treat the ADRs, improvement or resolution of the ADRs after drug discontinuation or dose reduction, and ADRs recurrence after the resumption of the suspected drug, and causality). The timeline for submitting the report depends on the seriousness and expectedness of the adverse reaction; unexpected or serious adverse reactions must be reported within 15 days. Particularly, adverse reactions resulting in death have to be reported immediately; all other adverse reactions have to be reported within 30 days [11].

### Data sources

The data were extracted from eligible ADRs reports submitted by our hospital to the CNCAM from January 1, 2010, to December 31, 2019; data on unreported ADRs were extracted from our Hospital Information System (HIS). The CNCAM, a network management information system under the Centre for Advanced Drug Monitoring [12] (http://www.adrs.org.cn/sso/login), is a subsidiary of the National Medical Products Administration and is responsible for collecting ADRs reported by healthcare facilities and corporations in China and monitoring ADRs to marketed drugs. It receives more than 1.5 million ADRs reports each year. At our hospital, ADRs data are submitted by clinical staff to the Department of Pharmacy and are then pooled by CPhs and uploaded to the CNCAM at the end of each month. In this study, we retrospectively analysed the changes in the number of inpatient ADRs reports submitted to the CNCAM per month, the number of unreported ADRs per month, and the standardized ADRs reporting rate per month after the implementation of the ADRs reporting system with CPhs intervention. Given that inpatients often have multiple underlying diseases and complex medications and often receive treatment via intravenous administration (which enables drugs to directly enter the blood stream) and that the intervention was based on the medications used by inpatients (to help clinicians to promptly report ADRs), we developed the following criteria for data extraction: (1) patients admitted to our hospital; (2) events between January 2010 to December 2019 (120 months); (3) ADRs reporting without CPhs intervention between January 2010 and May 2015 and ADRs reporting with CPhs intervention between June 2015 and December 2019.

### Outcome variable

In this study, the number of eligible ADR reports submitted by the CPhs per month, the number of unreported ARDs per month in the HIS, and the standardized ADRs reporting rate per month were used as continuous variables, and 9 years of data were extracted. Excel was used to establish a database for the ITS analysis. Statistical analysis and graphing were completed with R [13] (Version 4.1.0; The R Foundation for Statistical Computing Platform; Vienna, Austria). *P* < 0.05 was considered statistically significant. The R packages used included "Wats" [14], "lmtest" [15], "rio" [16], "scales" [17], "car" [18], “orcutt” [19], “Wooldridge” [20], "dplyr" [21], and "pander" [22]. This study was exempt from ethical review because we extracted only relevant (numeric) values and not personal or other information.

### Statistical analysis

ITS directly reflect changes in facts before and after clinical interventions, thereby effectively preventing bias and various confounding factors observed in other retrospective observational analyses. They represent a key research method for evaluating the outcome of clinical interventions [23–25]. They evaluate the immediate and potential long-term effects of any intervention by analysing the data before and after the intervention, including changes in the slope and trend. With segmented linear regression (SLR), the continuous time points before and after the intervention are used to fit the linear regression, and least squares estimation is used to calculate the SLR parameters of each time period to evaluate the outcome of the intervention [26]. Using multivariate linear regression, we retrospectively analysed the effect of the CPhs ADRs reporting intervention on the number of inpatient ADRs reports submitted to the CNCAM per month, the number of unreported ADRs per month, and the standardized ADRs reporting rate per month, as well as the trends in these outcomes. Compared with long-interval continuous values, short-interval continuous values are more likely to have similar adjacent values. Therefore, the Durbin-Watson (DW) test was performed to test the presence of continuous data, and the Cochrane-Orcutt regression was used to correct autocorrelations for data with first-order autocorrelations.

$${\varvec{Y}}{\varvec{t}}\boldsymbol{ }=\boldsymbol{ }{\varvec{\beta}}0\boldsymbol{ }+\boldsymbol{ }{\varvec{\beta}}1\boldsymbol{ }\times {\varvec{t}}{\varvec{i}}{\varvec{m}}{\varvec{e}}\boldsymbol{ }+\boldsymbol{ }{\varvec{\beta}}2\boldsymbol{ }\times {\varvec{i}}{\varvec{n}}{\varvec{t}}{\varvec{e}}{\varvec{r}}{\varvec{v}}{\varvec{e}}{\varvec{n}}{\varvec{t}}{\varvec{i}}{\varvec{o}}{\varvec{n}}\boldsymbol{ }+\boldsymbol{ }{\varvec{\beta}}3\boldsymbol{ }\times {\varvec{t}}{\varvec{i}}{\varvec{m}}{\varvec{e}}\boldsymbol{ }\ {\varvec{a}}{\varvec{f}}{\varvec{t}}{\varvec{e}}{\varvec{r}}\boldsymbol{ }\ {\varvec{i}}{\varvec{n}}{\varvec{t}}{\varvec{e}}{\varvec{r}}{\varvec{v}}{\varvec{e}}{\varvec{n}}{\varvec{t}}{\varvec{i}}{\varvec{o}}{\varvec{n}}\boldsymbol{ }+\boldsymbol{ }{\varvec{\varepsilon}}{\varvec{t}}$$

Model description: β0 represents the estimated value of Yt before the intervention; β1 represents the estimated value of the slope before the intervention; time represents the continuous variable of time; β2 represents the immediate effect after the intervention; intervention represents the start time of the CPhs ADRs reporting intervention; β3 represents the estimated change in Yt after the intervention; time after intervention represents the continuous time variable after the CPhs ADRs reporting intervention; and εt represents error, indicating any potential random change.

## Results

### DRC development

We retrieved a total of 218 eligible ADRs submitted by our hospital to the CNCAM over the past year. A total of 12 drugs were used as rescue drugs for ADRs. The situation of medication use was analysed and 81 single and combined medication regimens were extracted. We analyzed concomitant medications and selected 4 drugs that were used in various combinations (based on usage frequency) for inclusion in the DRC: dexamethasone (DEX), vitamin C (Vc), calcium gluconate (C.G.), and loratadine. The usage frequency and concomitant medications are shown in Table 1.

**Table 1:** Usage frequency of and concomitant medications used with rescue drugs for ADRs

	**Rescue medicine for ADR**	**DEX + Vc + C.G**	**DEX + Vc**	**DEX + C.G**	**Vc + C.G**	**DEX**	**Vc**	**C.G**	**Loratadine**	**total**
1	**Compound Aminophenazone and Barbital Injection**	**11**	**4**	**1**	**14**	**6**	**8**	**1**	**/**	**45**
2	**Epinephrine Injection**	**2**	**/**	**/**	**2**	**2**	**1**	**/**	**/**	**7**
3	**Promethazine Hydrochloride Injection**	**10**	**4**	**2**	**4**	**17**	**1**	**1**	**/**	**39**
4	**Compound Ammonium Glycyrrhizate Injection**	**4**	**3**	**/**	**2**	**/**	**3**	**/**	**/**	**12**
5	**Compound Glycyrrhizin Injection**	**24**	**4**	**/**	**11**	**1**	**4**	**1**	**/**	**45**
6	**Compound Glycyrrhizin Tablets**	**5**	**1**	**/**	**1**	**/**	**/**	**/**	**/**	**7**
7	**Mizolastine Sustained Release Tablets**	**/**	**/**	**/**	**/**	**/**	**/**	**/**	**/**	**0**
8	**Levocetirizine Dihydrochloride Tablets**	**8**	**2**	**1**	**10**	**/**	**4**	**/**	**/**	**25**
9	**Loratadine Tablets**	**12**	**1**	**/**	**10**	**3**	**3**	**/**	**9**	**38**

*DEX* Dexamethasone, *Vc* Vitamin C, *C.G.* Calcium gluconate

### ITS analysis

In this study, we collected a total of 8,113 ADRs reports made between January 2010 and December 2019, including inpatient ADRs reports submitted to the CNCAM and ADRs that were not reported to the CNCAM. Before the CPhs ADRs reporting intervention, approximately 7 (6.635) inpatient ADRs reports were submitted to the CNCAM per month, which was lower than the number of reports approximately 69 (69.293) after the intervention; approximately 45 (45.194) ADRs were unreported per month, which was higher than the number approximately 1 (1.103) after the intervention; the standardized ADRs reporting rate (per month) was 19.139%, which was lower than that after the intervention (82.773%).

#### Model 1: Number of inpatient ADRs reports submitted to the CNCAM per month was used as the dependent variable

**Fig. 1:**
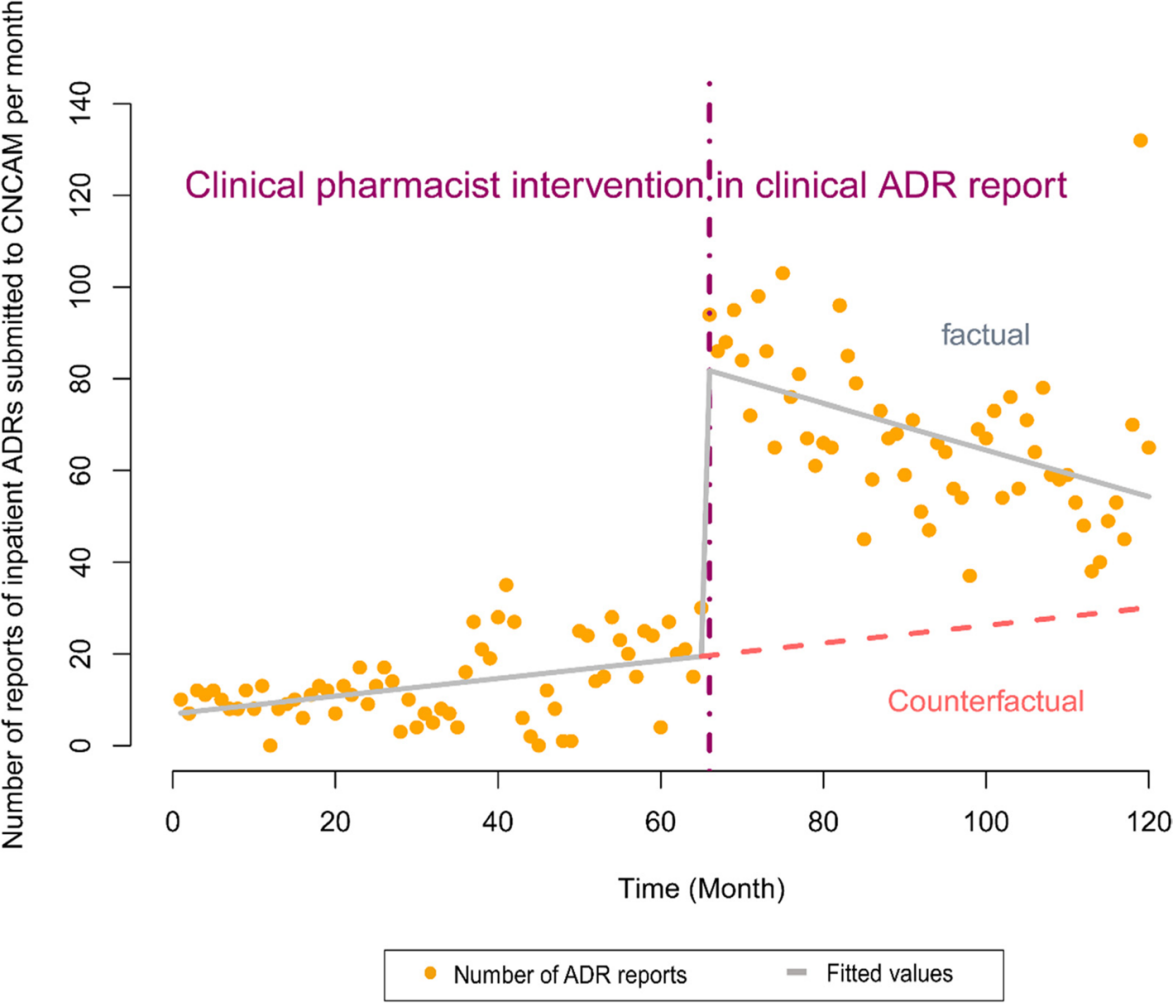
The trend in the number of inpatient ADRs reports submitted by our hospital to the CNCAM per month between January 2010 and December 2019. The vertical line represents the start time of the CPhs ADRs reporting intervention

In the model that used the number of inpatient ADRs reports submitted to the CNCAM per month as the dependent variable, the Cochrane-Orcutt regression was used to correct for data autocorrelations. The constant was 6.635 (*P* = 0.126912 > 0.05), and β1 was 0.198 (*P* = 0.078182 > 0.05), suggesting that the number of inpatient ADRs reports submitted to the CNCAM per month remained largely unchanged over time before the intervention. β2 was 62.658 (*P* < 0.01), suggesting that the number of inpatient ADRs reports submitted to the CNCAM increased by 63 ( 62.658) approximately during the first month of the intervention. β3 was -0.701 (*P* = 0.000151 < 0.01), indicating that the number of inpatient ADRs reports submitted to the CNCAM decreased by approximately 1 (0.701) per month starting at Month 2 of the intervention. The multiple R-squared was 0.7516, and the adjusted R-squared was 0.7451. The corrected Durbin-Watson statistic was 1.982263 (*P* = 0.3556 > 0.05), indicating that there was no data autocorrelation. Detailed information is shown in Table 2 and Fig. 1.

**Table 2:** Model 1 (the number of inpatient ADRs reports submitted to CNCAM per month was used as the dependent variable). Regression coefficients, standard errors, t-values, and *P*-values of the multivariate linear regression model used for the ITS segmented regression analysis

	Coefficients	Std. Error	*t*-value	*p*-value
β0	6.635	4.315	1.538	0.126912
β1	0.198	0.112	1.777	0.078182
β2	62.658	5.873	10.668	< 2.2e-16 ***
β3	-0.701	0.179	-3.920	0.000151 ***

Signif. codes: 0 ‘***’ 0.001 ‘**’ 0.01 ‘*’ 0.05 ‘.’

#### Model 2. Number of inpatient ADRs reports unreported to the CNCAM per month was used as the dependent variable

**Fig. 2:**
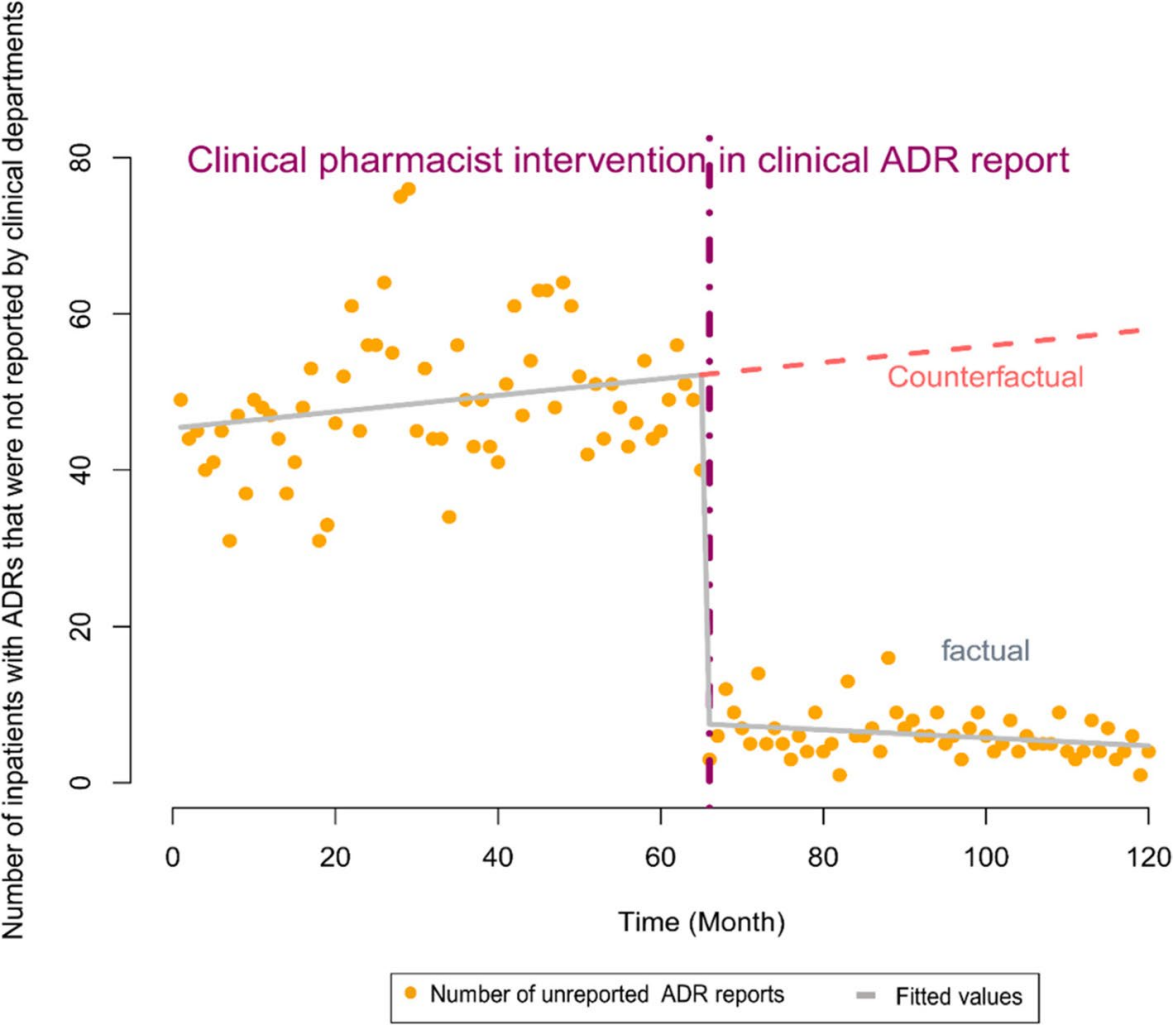
The trend in the number of unreported ADRs per month between January 2010 and December 2019. The vertical line represents the start time of the CPhs ADRs reporting intervention

In the model with the number of unreported ADRs per month as a dependent variable, the Cochrane-Orcutt regression was used to correct data autocorrelation. The constant was 45.194 (*P* < 0.01), and β1 was 0.105 (*P* = 0.1071 > 0.05), suggesting that the number of unreported ADRs per month remained largely unchanged before the intervention. β2 was -44.091 (*P* < 0.01), suggesting that the number of unreported ADRs decreased by 44 (44.091) approximately during the first month of the intervention. β3 was -0.167 (*P* = 0.1113 > 0.05), indicating that the number of unreported ADRs remained largely unchanged per month starting at Month 2 of the intervention. The multiple R-squared was 0.8387, and the adjusted R-squared was 0.8345. The corrected Durbin-Watson statistic was 2.06292 (*P* = 0.5294 > 0.05), indicating that there was no data autocorrelation. Detailed information is shown in Table 3 and Fig. 2.

**Table 3:** Model 2 (the number of unreported ADRs was used as a dependent variable). Regression coefficients, standard errors, t-values, and *P*-values of the multivariate linear regression model used in the ITS segmented regression analysis

	Coefficients	Std. Error	t-value	*p*-value
β0	45.194	2.514	17.979	< 2e-16 ***
β1	0.105	0.065	1.624	0.1071
β2	-44.091	3.381	-13.040	< 2e-16 ***
β3	-0.167	0.104	-1.605	0.1113

Signif. codes: 0 ‘***’ 0.001 ‘**’ 0.01 ‘*’ 0.05 ‘.’

#### Model 3: The standardized ADR reporting rate per month was used as a dependent variable

**Fig. 3:**
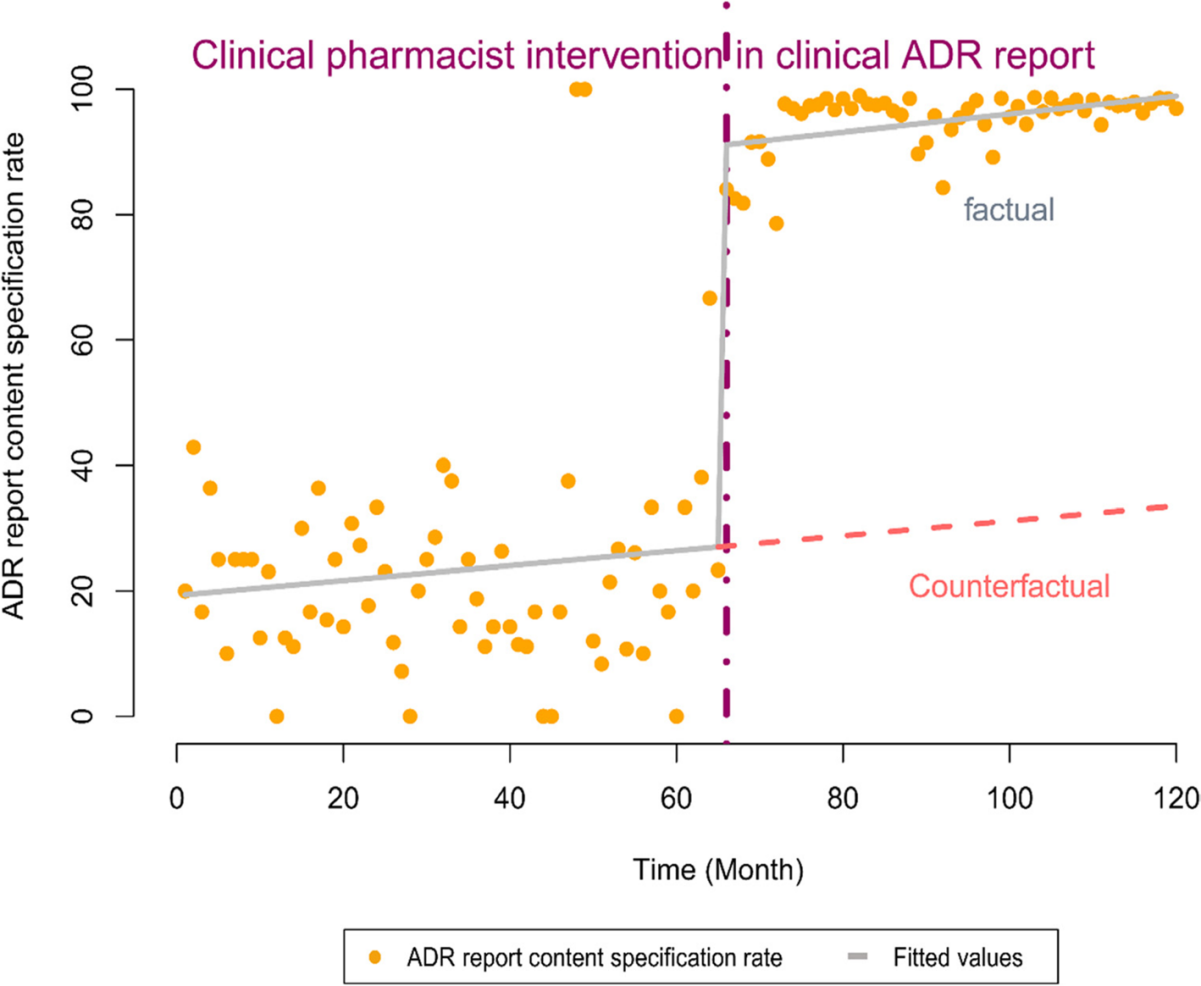
The trend in the standardized ADRs reporting rate (%) per month between January 2010 and December 2019. The vertical line represents the start time of the CPhs ADRs reporting intervention

In the model that used the standardized ADR reporting rate per month as a dependent variable, the Cochrane-Orcutt regression was used to correct data autocorrelation. The constant was 19.139 (*P* = 0.0005181 < 0.01), and β1 was 0.124 (*P* = 0.3681203 > 0.05), suggesting that the standardized ADRs reporting rate per month remained largely unchanged before the intervention. β2 was 63.634 (*P* < 0.01), indicating that the standardized ADR reporting rate increased by 63.634% during the first month of the intervention. β3 was 0.0226 (*P* = 0.9187487 > 0.05), indicating that the standardized ADR reporting rate per month remained largely unchanged starting at Month 2 of the intervention. Multiple R-squared was 0.7649, and adjusted R-squared was 0.7588. The corrected Durbin-Watson statistic was 1.85289 (*P* = 0.1397 > 0.05), indicating that there was no data autocorrelation. Detailed information is shown in Table 4 and Fig. 3.

**Table 4:** Model 3 (the standardized ADR reporting rate per month was used as a dependent variable). Regression coefficients, standard errors, t-values, and *P*-values of the multivariate linear regression model in the ITS segmented regression analysis

	Coefficients	Std. Error	*t*-value	*p*-value
β0	19.139	5.358	3.572	0.0005181 ***
β1	0.124	0.138	0.904	0.3681203
β2	63.634	7.094	8.970	6.48e-15 ***
β3	0.0226	0.221	0.102	0.9187487

Signif. codes: 0 ‘***’ 0.001 ‘**’ 0.01 ‘*’ 0.05 ‘.’

## Discussion

Using ITS, we retrospectively analyzed the effect of a CPhs ADRs reporting intervention on the number of inpatient ADR reports submitted to the CNCAM per month, the number of unreported ADRs per month, and the standardized ADR reporting rate per month in a Grade A, Class 3 hospital in China. Given that hospital patients are at risk for ADRs and that the team of CPhs developed a DRC that had hospital patients as the target population, we used hospital patients as the study group for data collection and excluded clinic patients and emergency patients, whose ARDs may not be promptly detected and/or reported. With ITS, data are segmented at equal intervals for processing and analysis. Therefore, ITS is one of the best statistical methods for retrospective analyses [23–25]. We extracted monthly data over a 9-year period and performed an ITS analysis to analyze the effect of the CPhs ADRs reporting intervention on the number of ADRs reports, the number of unreported ADRs, and the standardized ADRs reporting rate; additionally, we determined the long-term trends in these outcomes.

The number of inpatient ADRs reports submitted by our hospital to the CNCAM immediately increased 10.44 fold after the intervention, indicating a marked immediate effect of the intervention and highlighting the number of unreported ADRs. The number of inpatient ADRs reports submitted to the CNCAM per month declined slightly starting at Month 2, However, we consider that there may be multiple factors that induce this result. First, after feedback from physicians, it was considered that it might be related to avoidance of allergy-prone medications. That is, with the intervention of CPhs, clinicians are becoming more aware of reporting ADRs, and with the accumulation of experience in ADR reporting and medication use, physicians also try to avoid prescribing ADR-prone medications for their patients to reduce the risk of ADRs. Of course, this is from a few physician responses and further statistics on drug use are still needed. Second, it may be related to the timing of the intervention. This is because according to the current requirements in China, the time to submit an ADRs report depends on the severity and anticipation of the ADR. Thus some unexpected or serious ADRs must be reported within 15 days, those leading to death must be reported immediately, and others must be reported within 30 days, and this time accommodation may have led to a lag in reporting or nondetection of some ADRs, making the effect of the intervention decrease over time [11]。In addition, it has been suggested in the literature that regular training in the reporting of ADRs during the intervention phase of ongoing training can have a positive impact on the reporting of ADRs [27] Considering that the intervention of CPhs in this study did not add regular training for the medical population, this may be another reason for the decrease in reporting over time. Further analysis showed that the number of unreported ADRs decreased by approximately 40.94 fold during the first month of the CPhs intervention, and this number remained largely unchanged after Month 2, suggesting that the intervention effectively curbed ADR under-reporting. Furthermore, a focus on the quantity rather than the quality of ADRs reports may result in bias in subsequent pharmacovigilance studies based on ADRs reports. The standardized ADRs reporting rate (per month) was increased by 4.32 fold after the intervention. In summary, the CPhs ADRs reporting intervention played an active role in improving the number of ADRs reports and standardization. It should be mentioned, however, that this method was developed by the CPhs as a DRC based on hospital medication practices, which means that the CPhs' method has significantly improved the reporting of ADRs to some extent, but it is still not able to identify 100% of patients who may have ADRs (mainly those who present with ADRs but are not treated with medications), and there is no method or system available today that can identify 100% of ADRs. This is a limitation of the method and an important issue for CPhs to address.

ADRs are still a serious problem worldwide. Some studies show that elderly patients have become an at-risk population for ADRs [28]. ADRs increase the financial burden on patients and the healthcare system [29–31]. Furthermore, race, region, and lifestyle have major impacts on ADRs throughout the world [32]. ADRs reporting is very important as it enhances drug safety, facilitates post-market evaluation, and is the foundation for global pharmacovigilance studies [33]. However, under-reporting, delayed reporting, and incomplete reports remain common issues in ADRs reporting [34]. China has the largest population in the world, but its ADRs reporting rate is much lower than that of other countries. Comparing data from 15 countries from 2017 to 2018, China was unable to collect ADRs reported directly by consumers due to the lack of a consumer reporting system. The near-zero direct reporting rate by consumers cannot be compared to the 2.6% in Australia, 6.6.% in Belgium, 36% in Estonia, 21% in Finland, 16% in Germany, 9% in Greece, 36% in Ireland, 5% in Latvia Lithuania 12%, Portugal 5%, Slovakia 38.8%, and Spain 6.8% reporting rates [35]. In China, the spontaneous ADRs reporting model relies on healthcare facilities as primary reporters. Moreover, it relies on healthcare professionals’ awareness of ADRs to ensure patient safety and prompt reporting. A meta-analysis has shown that pharmacists can reduce the incidence of posthospital medication errors through guidance [36]. Our hospital is one of the highest-level hospitals in China. Our ADRs reporting situation is representative of that in other large general hospitals in China. In fact, in 2008, we launched a pharmacovigilance program. However, in this program, the CPhs were only responsible for uploading the reports submitted by the clinic and did not proactively establish methods to participate in the identification and quality management of ADRs. Therefore, in the 2008 ADRs report, due to a lack of effective management measures, only 93 ADRs were reported, of these, only 15 reports (16.13%) met the reporting standard, despite 45,169 admissions that year. These data and quality cannot reflect real-world ADRs. We understand that ADR reporting is critical to clinical research, and that the quality of clear, accurate, and detailed reporting is more likely to give a more precise orientation to medical research. In June 2015, a team of CPhs developed a DRC based on the clinical pattern of drug use and the principle of symptomatic care and then reviewed routine drug use in clinical practice. This method successfully improved the number of ADRs reports, underreporting, and standardization of ADRs reporting. In particular, in terms of improving the quality of reporting, the intervention of CPhs has led to a rapid increase in the standard rate of reporting the content of ADRs in a short period of time compared to before the involvement of CPhs and has maintained the subsequent reporting standards at a high level. These improvements have facilitated the monitoring of drug safety. However, it needs to be suggested that, considering that the statistics were performed on visible symptom ADRs in this study, further studies are still needed to observe whether the method is also effective in identifying ADRs without visible symptoms. In addition, this method can be used to establish a DRC that meets the local conditions according to the medication habits of medical institutions in different regions, so it has the characteristics of feasibility and good adaptability.

## Conclusion

This retrospective analysis shows that CPhs intervention in ADRs reporting significantly improves the number of ADRs reports, reduces nonreporting, and improves the standardization of ADRs reports, indicating that CPhs play an active role in ADRs reporting. However, this method has some limitations. As a method to improve ADR identification, it enables effective identification of ADRs that are present and underreported to alert CPhs for timely reporting of ADRs, but not for patients with unmedicated ADRs. ADRs are still considered unpredictable worldwide, and ADRs-predicting systems and software are lacking [37]. Researchers are working on systems for effective ADRs detection and reporting, but they have yet to identify methods for predicting ADRs. We realized during data collection in this study that early adverse reactions without obvious symptoms are difficult to detect. For this reason, on the basis of ADRs reported over the 9 years, we plan to investigate how to predict ADRs based on unusual fluctuations in clinical indicators such as platelets, hemoglobin, albumin, liver function, and kidney function. The goal is to identify potential ADRs to provide early warnings. We will report the results in due time.

## Data Availability

All data generated or analysed during this study are included in this published article. For non-commercial use, please contact the corresponding author to obtain the data of this paper.

## Abbreviations

ADRs: Adverse drug reactions
C.G.: Calcium gluconate
CNCAM: The China National Centre for Adverse Drug Reaction Monitoring
CPhs: Clinical pharmacists
DEX: Dexamethasone
DRC: Drug review catalogue
DW test: Durbin-Watson test
HIS: Hospital Information System
ITS: Interrupted time series
SLR: Segmented linear regression
SRS: The spovntaneous reporting system
Vc: Vitamin C
